# INVERSE RELATIONSHIP BETWEEN PLASMA PROTEINS AND SATISFACTORY SURGICAL WOUNDS OUTCOME

**DOI:** 10.1590/1413-785220233102e263313

**Published:** 2023-06-09

**Authors:** PEDRO AUGUSTO JAQUETO, IASMIN CHAGAS SABBAG, LEONARDO PEDRÃO DA SILVA, LUCCAS FRANCO BETTENCOURT NUNES, CARLOS AUGUSTO DE MATTOS, VÂNIA APARECIDA LEANDRO-MERHI

**Affiliations:** 1. Pontifícia Universidade Católica de Campinas (PUC-Campinas), Postgraduated Program in Health Sciences, Campinas, SP, Brazil.; 2. Hospital da PUC-Campinas, Orthopedics and Traumatology, Campinas, SP, Brazil.; 3. Hospital da PUC-Campinas, Arthroscopy and Sports Trauma Group, Campinas, SP, Brazil.

**Keywords:** Osteosynthesis, Fracture, Postoperative Complications, Surgical Wound, Albumin, Transferrin, Osteossíntese, Complicações Pós-Operatórias, Ferida Cirúrgica, Albumina, Transferrina

## Abstract

**Objective:**

This study investigated the factors associated with satisfactory early postoperative wound conditions.

**Method:**

A prospective study was conducted with patients (n=179) submitted to osteosynthesis in general, in a hospital orthopedics service. In the preoperative period, patients underwent laboratory exams and the surgical indications were based on the type of fracture and the patient’s clinical conditions. In the postoperative period, patients were evaluated based on the presence of complications and considering their surgical wounds. Chi-square, Fisher, Mann-Whitney, and Kruskal-Wallis tests were used in the analysis. To identify the factors associated with wound condition, univariate and multiple logistic regression analysis was used.

**Results:**

In the univariate analysis, each transferring unit reduction increased the chance of satisfactory outcome by 1.1% (p=0.0306; OR= 0.989 (1.011); 95%CI= 0.978;0.999; 1.001;1.023). The presence of SAH increased 2.7 fold the chance of satisfactory outcome (p=0.0424; OR= 2,667; 95%CI= 1,034;6,877). Hip fracture increased 2.6 fold the chance of satisfactory outcome (p=0.0272; OR=2.593; IC95%=1.113; 6.039). And the absence of a compound fracture increased 5.5 fold the chance of satisfactory wound outcome (p=0.0004; OR=5,493; 95%CI=2,132;14,149). In the multiple analysis, patients with non compound fractures were 9.7 times more likely to experience a satisfactory outcome when compared to patients with compound fractures (p=0.0014; OR=9,687; 95%CI= 2,399; 39,125).

**Conclusion:**

There was an inverse relationship between plasma proteins levels and satisfactory surgical wounds outcome. Only exposure remained associated with wound conditions. Level Of Evidence: II, Prospective Study.

## RESUMO

**Objetivo:**

Este estudo investigou os fatores associados à condição satisfatória de ferida pós-operatória precoce.

**Método:**

Foi desenvolvido um estudo prospectivo com pacientes (n=179) submetidos a osteossínteses em geral, em um serviço de ortopedia hospitalar. No período pré-operatório os pacientes foram submetidos a exames laboratoriais e as indicações cirúrgicas foram baseadas no tipo de fratura e condições clínicas. No período pós-operatório, os pacientes foram avaliados conforme a presença de complicações e feridas operatórias. Foram utilizados na análise os testes Qui-quadrado, Fisher, Mann-Whitney, Kruskal-Wallis. Para identificar os fatores associados à condição da ferida, foi utilizada a análise de regressão logística univariada e múltipla.

**Resultados:**

Na análise univariada, cada unidade a menos de transferrina, aumentou a chance de condição satisfatória em 1.1% (p= 0.0306; OR= 0.989 (1.011); IC95%= 0.978;0.999; 1.001;1.023). A presença de HAS aumentou a chance de condição satisfatória em 2.7 vezes (p=0.0424; OR= 2.667; IC95%= 1.034;6.877). Fratura de quadril aumentou a chance de condição satisfatória em 2.6 vezes (p=0.0272; OR=2.593; IC95%=1.113;6.039). E não apresentar fratura exposta, aumentou a chance de condição satisfatória da ferida em 5.5 vezes (p=0.0004; OR=5.493; IC95%=2.132;14.149). Na análise múltipla, fraturas sem exposição, apresentaram chance 9.7 vezes maior de apresentar condição satisfatória do que insatisfatória; quando comparadas às que apresentaram exposição (p=0.0014; OR=9.687; IC95%= 2,399; 39.125).

**Conclusão:**

Houve uma relação inversa entre proteínas plasmáticas e presença de feridas operatórias satisfatórias. Apenas a exposição se manteve associada à condição da ferida. Nível de Evidência: II; Estudo Prospectivo.

## INTRODUCTION

Surgical site complications are the most feared complications after those of osteosynthesis, as observed in a systematic review and meta-analysis performed by Shao *et al* , 2017.^1^ As reported in a study by Renz *et al* , 2017^2^ infection rates of 1 to 5% occur in the case of closed fractures osteosynthesis and up to 30% in compound fractures, making the diagnosis of infections associated with osteosynthesis challenging.^2^

The incidence of peri-implant infections may vary according to the wound site, patient’s age, gender, race and existing comorbidities, such as diabetes, obesity or rheumatoid arthritis^3 , 4^ and a large increase in demand for hip and knee arthroplasties is estimated by the year 2030.^3^ In a hospital-based case-control study^4^ that evaluated the risk factors for infections in hip and knee arthroplasties, logistic regression analysis indicated that patients with diabetes, advanced age, body mass index (BMI) ≥28 kg/m^2^ and alcohol abuse or living in rural areas were at increased risk of periprosthetic joint infection.^4^

In a study that evaluated the relative risk of postoperative mortality and periprosthetic joint infection associated with comorbidities in patients undergoing total knee arthroplasty (TKA),^5^ the authors showed that the independent risk factors for postoperative mortality at 90 days were congestive heart failure, metastatic cancer, kidney disease, peripheral vascular disease, cerebrovascular disease, lymphoma, cardiac arrhythmia, dementia, pulmonary circulation disorders, and chronic liver disease. In the same study^5^ it was found that independent risk factors for periprosthetic joint infections were congestive heart failure, chronic pulmonary disease, preoperative anemia, diabetes, depression, renal disease, pulmonary circulation disorders, obesity, among others.^5^

Blood glucose control is an important factor in preventing surgical site infection and some studies indicate a correlation between obesity, hyperglycemia, diabetes and increased risk of osteosynthesis and arthroplasties infections.^6 , 7^

Other investigations point to an important association of serological malnutrition with postoperative wound complications.^8^ Both malnutrition and obesity could favor postoperative complications;^9^ malnutrition additionally could favor late infections.^10^

Other findings in the literature indicate that malnutrition diagnosed by albumin, transferring levels and total lymphocyte count could be associated with postoperative complications, thus suggesting the need for further investigations into the predictive value of these indices in the evaluation of postoperative complications.^11^

In view of the above, the present study investigated factors associated with early satisfactory postoperative wound condition in patients in the process of fracture osteosynthesis in general.

## METHOD

### Study Design, Participants, ethical approval and inclusion and exclusion criteria

The study was prospective, performed with patients undergoing osteosynthesis procedures in general, in a hospital orthopedics service of a Public Health Service-SUS. The study started after approval by the Institution’s Ethics and Research Committee and the signing of the Free and Informed Consent Form (FICF).

All patients in the process of osteosynthesis in general, in elective or urgent surgeries, in a period of 6 months during the year 2021 were included. Patients requiring intensive pre-surgical support and compensation of the clinical condition for elective surgery were excluded. After reviewing the inclusion and exclusion criteria, one hundred and seventy-nine (n=179) patients were considered for this study.

### Methodological procedures

Patients were admitted to the Hospital Emergency Care Service and came on their own or were brought in by rescue teams. They were screened and attended by the orthopedic team, who identified the fractures and the need for emergency surgery or elective surgery. In the preoperative period, all patients underwent laboratory tests and surgical indications were based on the type of fracture and the patients’ clinical conditions. In the postoperative period, patients were evaluated according to the presence of wound complications. After the surgery and hospital discharge, patients returned to the outpatient clinic for post-surgical evaluation and for evaluation of the operative wounds conditions (satisfactory and unsatisfactory). The same preoperative, intraoperative and postoperative procedures were used, with patients who were discharged between 24 and 48 hours from admission. The variables assessed were:

- Demographic and clinical data:- data such as gender, age, presence of comorbidities, even if mild or controlled, such as diabetes mellitus, Systemic Arterial Hypertension (SAH), alcohol abuse and smoking were collected.
- Patient-reported body weight and height upon admission and BMI was calculated.^12 , 13^
- c. Laboratory tests: complete blood count, coagulogram, electrolytes and plasma proteins. Nutritional status was assessed based on plasma proteins levels such as albumin’s and transferrin’s. Plasma albumin levels <3.5 g/dl and transferrin <200 mg/dl were considered to reflect a malnourishment condition.^14^
- Osteosynthesis surgeries were performed according to the fracture that occurred and according to the techniques described in the AO.^15^
- Surgical and other variables:- the occurrence of intraoperative complications, amputations, neurological and vascular injuries were assessed intraoperatively. The occurrence of postoperative cardiovascular reactions was also assessed.

This project was approved by the Pontifical Catholic University of Campinas-SP-Brazil’s Ethics Committee (reference number:- 5.182.817).

### Statistical Analysis

A descriptive analysis of the variables was performed to characterize the population, with frequency data for the categorical variables and position and dispersion measure for the continuous variables. To compare proportions, the chi-square or Fisher’s exact test was used, when necessary. To compare continuous or sortable measures between two groups, the Mann-Whitney test was applied and the Kruskal-Wallis test was applied among three groups, followed by Dunn’s test, when necessary. To identify the factors associated with wound condition, univariate and multiple logistic regression analysis were used. The variable selection process used was stepwise. The significance level adopted for the tests was 5%.^16 , 17 , 18^

## RESULTS

The mean age of the population (n=179) was 47.87±24.41 years (median=48); 36.3% (n=65) were female and 63.7% (n=114) were male. The age groups were 10.1% (n=18) between 0 and 18 years of age; 62.6% (n=112) between 19 and 65 years old and 27.4% (n=49) were aged over 65 years. In this series, 67.3% (n=115) had no comorbidities, 18.7% (n=32) had only one comorbidity and 14% (n=24) had two to four comorbidities. The most frequent comorbidities were diabetes mellitus (10.5%, n=18); SAH (25.7%, n=44); smoking (8.2%, n=14) and alcohol abuse (5.8%, n=10). The wound condition 7 days postoperatively was considered satisfactory in 70.8% (n=121) and unsatisfactory in 29.2% (n=50). By BMI, 59.7% (n=86) were overweight, 31.9% (n=46) had adequate body weight and 8.3% (n=12) were underweight. As for the fractures site, 72.6% (n=130) had fractures of the lower limbs and 27.4% (n=49) of the upper limbs. A total of 30.2% (n=54) patients had hip fractures, involving acetabulum=3.7% (n=2), femoral neck=13.0%(n=7), femoral diaphyseal=1.9% (n=1), periprosthetic hip fracture=1.9% (n=1) and transtrochanteric femoral fracture = 79.6% (n=43). Compound fractures were observed in 12.3% (n=22) patients while 87.7% (n=157) did not present with open fractures.

Women were older (p<0.0001), and a higher percentage had diabetes mellitus (p=0.0030), cardiovascular diseases (p=0.0010), SAH (p=0.0229), comorbidities in general (p=0.0001) and a higher percentage of hip fracture (p=0.0004). Men had a higher percentage of preoperative complications (p=0.0277).

The age group between 0-18 years had lower BMI (p=0.0026), higher percentage of BMI adequacy (p<0.0001), lower blood glucose (p=0.0256), higher albumin (p=0.0004) (higher percentage within the normal values), without comorbidities (p<0.0001), higher percentage of International Normalized Ratio (INR) above normal (p=0.0347) and higher percentage of transferrin within normality (p=0.0004).

The age group between 19-65 years had higher BMI (p=0.0313) (higher percentage of overweight and obesity), low frequency of comorbidities (p<0.0001), higher percentage of lower limb fractures (p=0.0435) and with open fracture (p=0.0292), higher percentage of preoperative complications (p=0.0074), higher percentages of hemoglobin (p=0.0056) and hematocrit within the normal range (p=0.0060).

The age group above 65 years exhibited low weight by BMI classified by age (p<0.0001), lower values of hemoglobin (p<0.0001), hematocrit (p=0.0002), platelets (p=0.0197), International Normalized Ratio (INR) (p=0.0347), albumin (p=0.0004) and transferrin (p=0.0111). There were even higher values of blood glucose (p=0.0256), hemoglobin (p=0.0056), hematocrit (p=0.0060), International Normalized Ratio (INR) below normal values (p=0.0347), higher percentage of protein depletion by albumin (p=0.0154) and transferrin (p=0.0004). Higher percentages of diabetes mellitus (p<0.0001), cardiovascular diseases (p<0.0001), comorbidities in general (p<0.0001), lower limb fractures (p=0.0435) and hip (p<0.0001), also were more observed in this age group.

Wounds with satisfactory conditions at 7 days were characterized by lower transferring values (p=0.0440), albumin below the reference (p=0.0463), without preoperative complications (p=0.0001), without open fracture (p=0.0001), hip fractures (p=0.0239) and patients with SAH (p=0.0372). ( Table 1 )

**Table 1:** Descriptive analysis and comparisons of the variables studied with the wound condition 7 days postoperatively (N=179).

Variables	Category	Unsatisfactory Wound Condition N= 50	Satisfactory Wound Condition N= 121	P-value
Age (years)	X±DP	42.58 ± 21.55	48.37 ± 24.49	0.2070^1^
Body mass index	X±DP	26.30 ± 2.96	26.22 ± 3.32	0.7365^1^
Number of comorbidities	X±DP	0.27 ± 0.57	0.54 ± 0.89	0.0768^1^
Hemoglobin	X±DP	13.25 ± 2.02	12.80 ± 2.02	0.2787^1^
Hematocrit	X±DP	38.63 ± 6.13	38.07 ± 5.77	0.7808^1^
Leukocytes	X±DP	11854.22 ± 5475.24	11959.37 ± 12349.28	0.5162^1^
APTT	X±DP	28.39 ± 4.18	28.33 ± 5.06	0.9654
INR	X±DP	1.01 ± 0.10	1.04 ± 0.14	0.2709^1^
Platelets	X±DP	214973.64 ± 90480.86	232412.27 ± 77547.05	0.6980^1^
Blood glucose	X±DP	124.09 ± 32.85	118.65 ± 30.73	0.4462^1^
Albumin	X±DP	4.07 ± 0.51	3.85 ± 0.63	0.3284^1^
Transferrin	X±DP	251.19 ± 37.93	222.60 ± 58.08	0.0440^1^
Gender				
female	N (%)	13 (26.0%)	48 (39.7%)	0.0896^2^
male	N (%)	37 (74.0%)	73 (60.3%)	
Age Group (years)				
up to 18 years	N (%)	5 (10.0%)	13 (10.7%)	0.1830^2^
19-65 years	N (%)	37 (74.0%)	73 (60.3%)	
> 65 years	N (%)	8 (16.0%)	35 (28.9%)	
Classified body mass index				
overweight	N (%)	25 (65.8%)	59 (59.6%)	0.6494^2^
adequate	N (%)	10 (26.3%)	34 (34.3%)	
underweight	N (%)	3 (7.9%)	6 (6.1%)	
Diabetes				
no	N (%)	45 (93.8%)	103 (88.8%)	0.4004^3^
yes	N (%)	3 (6.3%)	13 (11.2%)	
Smoking				
no	N (%)	47 (97.9%)	106 (91.4%)	0.1780^3^
yes	N (%)	1 (2.1%)	10 (8.6%)	
Alcoholism				
no	N (%)	46 (95.8%)	110 (94.8%)	1.0000^3^
yes	N (%)	2 (4.2%)	6 (5.2%)	
Hypertension				
no	N (%)	42 (87.5%)	84 (72.4%)	0.0372^2^
yes	N (%)	6 (12.5%)	32 (27.6%)	
Presence of comorbidities				
no	N (%)	38 (79.2%)	77 (66.4%)	0.1036^2^
yes	N (%)	10 (20.8%)	39 (33.6%)	
Number of comorbidities				
0	N (%)	38 (79.2%)	77 (66.4%)	0.1906^2^
1	N (%)	7 (14.6%)	21 (18.1%)	
2-4	N (%)	3 (6.3%)	18 (15.5%)	
Limb				
lower	N (%)	33 (66.0%)	89 (73.6%)	0.3204^2^
upper	N (%)	17 (34.0%)	32 (26.4%)	
Hip				
no	N (%)	42 (84.0%)	81 (66.9%)	0.0239^2^
yes	N (%)	8 (16.0%)	40 (33.1%)	
Compound fracture				
no	N (%)	36 (72.0%)	113 (93.4%)	0.0001^2^
yes	N (%)	14 (28.0%)	8 (6.6%)	
Osteosynthesis				
no	N (%)	21 (42.0%)	46 (38.0%)	0.6274^2^
yes	N (%)	29 (58.0%)	75 (62.0%)	
Preoperative complications				
soft parts	N (%)	3 (6.0%)	6 (5.0%)	0.0001^2^
exposed soft parts	N (%)	17 (34.0%)	10 (8.3%)	
no complications	N (%)	30 (60.0%)	105 (86.8%)	
Hemoglobin				
low	N (%)	17 (37.0%)	43 (36.1%)	0.9216^2^
normal	N (%)	29 (63.0%)	76 (63.9%)	
Hematocrit				
low	N (%)	21 (45.7%)	50 (42.4%)	0.7034^2^
normal	N (%)	25 (54.3%)	68 (57.6%)	
Leucocytes				
low	N (%)	23 (51.1%)	57 (50.4%)	0.9395^2^
normal	N (%)	22 (48.9%)	56 (49.6%)	
APTT				
low	N (%)	6 (13.3%)	19 (16.4%)	0.6320^2^
normal	N (%)	39 (86.7%)	97 (83.6%)	
INR				
low	N (%)	5 (10.9%)	9 (7.6%)	0.7992^2^
above	N (%)	7 (15.2%)	19 (16.1%)	
normal	N (%)	34 (73.9%)	90 (76.3%)	
Platelets				
low	N (%)	7 (15.9%)	16 (14.5%)	0.8302^2^
normal	N (%)	37 (84.1%)	94 (85.5%)	
Blood glucose				
<100	N (%)	4 (17.4%)	19 (34.5%)	0.2932^2^
100-125	N (%)	10 (43.5%)	17 (30.9%)	
>125	N (%)	9 (39.1%)	19 (34.5%)	
Albumin				
low	N (%)	3 (10.7%)	17 (30.4%)	0.0463^2^
normal	N (%)	25 (89.3%)	39 (69.6%)	
Transferrin				
low	N (%)	4 (15.4%)	17 (32.1%)	0.1146^2^
normal	N (%)	22 (84.6%)	36 (67.9%)	

INR:- International Normalized Ratio. APTT:- Activated Partial Thromboplastin Time. ^1^ Mann-Whitney test; ^2^ Chi-square test; ^3^ Fisher’s exact test.

In the univariate analysis, each unit less in the transferrin level increased the chance of satisfactory condition by 1.1%. *(p=0.0306; OR=0.989 (1.011); 95%CI= 0.978;0.999; 1.001; 1.023).* The presence of SAH increased the chance of satisfactory outcome by 2.7 times *(p=0.0424; OR= 2,667; 95%CI= 1,034;6,877)* . Hip fracture increased the chance of satisfactory outcome by 2.6 times *(p=0.0272; OR=2,593; 95%CI=1,113; 6,039* ). And absence of open fracture, increased the chance of satisfactory wound outcome by 5.5 times *(p=0.0004; OR=5.493; 95%CI=2.132; 14.149)* . In our series, only compound fractures remained associated with wound condition. Closed fractures patients were 9.7 times more likely to present a satisfactory outcome when compared to those with compound fractures *(p=0.0014; OR=9.687; 95%CI= 2.399; 39.125* ). ( Table 2 )

**Table 2:** Univariate and multiple logistic regression analysis for the study of factors associated with wound condition at 7 days.

Variables	Category	p-value	OR	95% CI
**Univariate analysis ^1^**				
Age		0.1478	1.011	0.996;1.025
BMI	adequate vs overweight	0.3978	1.441	0.618;3.357
	underweight vs overweight	0.8244	0.847	0.196;3.659
Albumin		0.1271	0.539	0.243;1.192
Transferrin		0.0306	0.989 (1.011)	0.978;0.999 (1.001;1.023)
Diabetes	no vs yes	0.3371	0.528	0.143;1.945
Smoking	no vs yes	0.1613	0.226	0.028;1.813
Alcoholism	no vs yes	0.7860	0.797	0.155;4.096
Arterial hypertension	yes x no	0.0424	2.667	1.034;6.877
Number of comorbidities	0 vs 2-4	0.0971	0.338	0.094;1.218
	1 vs 2-4	0.3625	0.500	0.112;2.223
Limb	LL vs UL	0.3215	1.433	0.704;2.917
Hip	yes vs no	0.0272	2.593	1.113;6.039
Compound fracture	no vs yes	0.0004	5.493	2.132;14.149
Osteosynthesis	yes vs no	0.6276	1.181	0.604;2.309
Blood glucose	normal vs above	0.1375	2.507	0.745;8.432
Albumin	below vs normal	0.0566	3.632	0.964;13.680
Transferrin	below vs normal	0.1226	2.597	0.773;8.722
**Multiple analysis ^2^**				
Compound fracture	no vs yes	0.0014	9.687	2.399; 39.125

BMI: body mass index. LL: lower limb. UL:-upper limb. ^1^ Univariate analysis: modeling the probability of satisfactory condition. Univariate logistic regression; OR=odds ratio, 95% CI (95% confidence interval for OR). ^2^ Multiple Analysis:- stepwise variable selection process.

There was a statistical difference for the number of comorbidities (p=0.0066), presence of SAH (p=0.0473), presence of comorbidities (p=0.0015) and number of grouped comorbidities (p=0.0046 ), compared with serum albumin levels. ( Table 3 )

**Table 3:** Descriptive analysis and comparison of comorbidities with serum albumin.

Variables/ Comorbidities	Category	Albumin * Low (n=20)	Albumin * Normal (n=68)	p-value
Number of comorbidities	X±DP	1.11 ± 0.81	0.63 ± 1.08	0.0066^1^
**Diabetes**				
No	N (%)	13 (68.4%)	56 (87.5%)	0.0778^3^
Yes	N (%)	6 (31.6%)	8 (12.5%)	
**Smoking**				
No	N (%)	17 (89.5%)	56 (87.5%)	1.0000^3^
Yes	N (%)	2 (10.5%)	8 (12.5%)	
**Alcoholism**				
No	N (%)	16 (84.2%)	60 (93.8%)	0.1933^3^
Yes	N (%)	3 (15.8%)	4 (6.3%)	
**Arterial hypertension**				
No	N (%)	9 (47.4%)	46 (71.9%)	0.0473^2^
Yes	N (%)	10 (52.6%)	18 (28.1%)	
**Comorbidities**				
No	N (%)	5 (26.3%)	43 (67.2%)	0.0015^2^
Yes	N (%)	14 (73.7%)	21 (32.8%)	
**Number of comorbidities grouped**				
0	N (%)	5 (26.3%)	43 (67.2%)	0.0046^3^
1	N (%)	7 (36.8%)	10 (15.6%)	
2-4	N (%)	7 (36.8%)	11 (17.2%)	

*Albumin. ^1^ Mann-Whitney test; ^2^ Chi-square test; ^3^ Fisher›s exact test.

There was also a statistical difference for the number of comorbidities (p=0.0161), presence of SAH (p=0.0090), presence of comorbidities (p=0.0081) and number of grouped comorbidities (p=0.0310 ), compared with serum transferrin levels. ( Table 4 )

**Table 4:** Descriptive analysis and comparison of comorbidities with serum transferrin.

Variables/ Comorbidities	Category	Transferrin* Below (n=21)	Transferrin* Normal (n=61)	p-value
Number of comorbidities	X±DP	1.16 ± 1.07	0.61 ± 1.00	0.0161^1^
**Diabetes**				
No	N (%)	13 (68.4%)	51 (86.4%)	0.0924^3^
Yes	N (%)	6 (31.6%)	8 (13.6%)	
**Smoking**				
No	N (%)	17 (89.5%)	52 (88.1%)	1.0000^3^
Yes	N (%)	2 (10.5%)	7 (11.9%)	
**Alcoholism**				
No	N (%)	17 (89.5%)	54 (91.5%)	1.0000^3^
Yes	N (%)	2 (10.5%)	5 (8.5%)	
**Arterial hypertension**				
No	N (%)	8 (42.1%)	44 (74.6%)	0.0090^2^
Yes	N (%)	11 (57.9%)	15 (25.4%)	
**Comorbidities**				
No	N (%)	6 (31.6%)	39 (66.1%)	0.0081^2^
Yes	N (%)	13 (68.4%)	20 (33.9%)	
**Number of comorbidities grouped**				
0	N (%)	6 (31.6%)	39 (66.1%)	0.0310^3^
1	N (%)	6 (31.6%)	9 (15.3%)	
2-4	N (%)	7 (36.8%)	11 (18.6%)	

*Transferrin. ^1^ Mann-Whitney test; ^2^ Chi-square test; ^3^ Fisher›s exact *test.*

**Figure 1:**
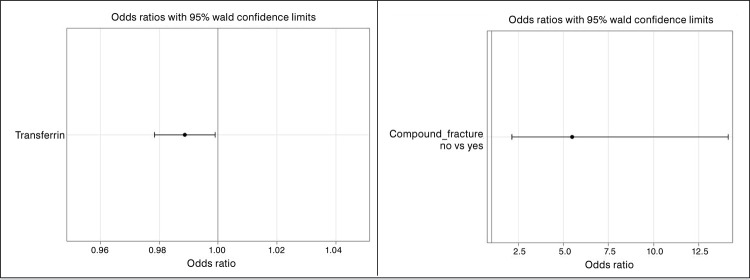
Odds ratios and relevant 95% confidence intervals for the main factors associated with satisfactory wound condition after 7 days.

The odds ratios and relevant 95% confidence intervals for the main factors associated with satisfactory wound condition after 7 days are reported in Figure 1 .

## DISCUSSION

The purpose of this investigation was to assess the risk factors associated with postoperative complications in patients undergoing fracture osteosynthesis process in general. The findings in this investigation pointed to several controversies, observed in previous studies.^9 , 10^ In our series, contradictory outcomes were found in connection to plasma proteins and their association with postoperative wounds, showing that reduced transferrin plasma levels increased the chances of patients presenting satisfactory wound outcome; these findings are opposed to those found in other investigations reported in the literature.^8 , 9 , 10 , 11^ These findings suggest the need for further investigations, probably with a larger sample size, multicenter studies and in normal times in emergency room and hospital emergency room visits, considering that our study was conducted in a period of full blown Covid 19 pandemic. Another factor that deserves to be highlighted in the present investigation refers to the fact that the presence of SAH was associated with a satisfactory wound outcome, a factor that could suggest or raise a hypothesis that these patients were undergoing treatment for the disease (SAH). However, as this issue was not the object of this study, these considerations cannot be confirmed in the present investigation. Another finding observed in our study was the fact that patients with hip fractures and without open fractures presented satisfactory wound outcomes, as well as patients with lower levels of albumin and transferrin and without preoperative complications. Another relevant finding is that there was a significant association between a greater number of comorbidities and lower than normal levels of albumin and transferrin. Our data also showed an association between the presence of hypertension and lower than normal levels of albumin and transferrin. Finally, in our sample, in our orthopedic hospital service, which serves a representative population of a large metropolitan region, only compound fractures were associated with the wound outcome.

Unlike the findings in this investigation, in a study^14^ that evaluated the relationship between some nutritional parameters and the development of periprosthetic joint infection in patients undergoing total joint arthroplasty, the authors showed through multivariate analysis that lower levels of albumin and hemoglobin, were significantly associated with periprosthetic joint infection.^14^ In this same study, the authors observed that albumin had the highest specificity and positive predictive value compared to all the other markers investigated.^14^ Other relevant findings, different from the results observed in the present study, show interesting data; pointing out that hypoalbuminemia could be used as a preoperative predictor of outcomes and the understanding of the effects of malnutrition on perioperative complications could guide surgical interventions.^10^

Another study suggested that the investigation of nutritional status should not be neglected when considering total joint arthroplasty in patients with low body weight,^9^ and that even severe obesity may not be associated with a higher risk of death after arthroplasty.^9^

There are also investigations showing the incidence of surgical site infection in a retrospective study with patients undergoing primary joint arthroplasty, pointing out that plasma serum albumin levels below 36.7g/L, BMI≥28 and ASA ≥3 were considered risk factors of postoperative wound infection.^19^ Deep wound infections in patients undergoing knee replacements were also observed in those with reduced albumin and transferring levels and lymphocyte counts.^20^

The findings of our work suggest that it may not be relevant to require plasma protein tests in patients with this type of trauma in emergency care and hospital emergency services. Perhaps in other clinical and surgical situations, this requirement would be relevant. But it is important to highlight the fundamental role of the nutritional status in optimizing clinical and surgical outcomes, and in preventing postoperative infections and wound complications, as observed in another investigation that showed that albumin, prealbumin and transferring levels may be predictive of wound complications after total knee arthroplasty.^21^ In the present study, we used linear regression analysis to assess changes over time and highlighted the fundamental role of nutritional investigation.^21^

It is also important to note that the present study was conducted at a referral service for traffic accidents and we believe that most men suffered fractures of the lower limbs not related to the hip, as they were victims of traffic accidents, and most women had hip fractures, probably related to osteoporosis. It is necessary that these findings be further investigated in future research, perhaps with a larger sample and reproduced in other research centers.

### Study limitations

The small sample size can be considered the main limiting factor of this investigation. Another limiting factor can be attributed to the issue of data collection in a hospital emergency service and emergency room, which may have influenced the findings of albumin and transferrin. Another limiting factor refers to the fact that this work was conducted during the full blown period of Covid-19 pandemic.

## CONCLUSION

In this series, the findings allowed us to conclude that there was an inverse relationship between plasma proteins and surgical wounds outcome considered satisfactory. Only compound fractures were associated with wound outcome.
